# The potential impact of AI innovations on US occupations

**DOI:** 10.1093/pnasnexus/pgae320

**Published:** 2024-09-24

**Authors:** Ali Akbar Septiandri, Marios Constantinides, Daniele Quercia

**Affiliations:** Nokia Bell Labs, Cambridge CB3 0FA, United Kingdom; Nokia Bell Labs, Cambridge CB3 0FA, United Kingdom; Nokia Bell Labs, Cambridge CB3 0FA, United Kingdom; King’s College London, London WC2R 2LS, United Kingdom

**Keywords:** future of work, AI, patents, labor market

## Abstract

An occupation is comprised of interconnected tasks, and it is these tasks, not occupations themselves, that are affected by Artificial Intelligence (AI). To evaluate how tasks may be impacted, previous approaches utilized manual annotations or coarse-grained matching. Leveraging recent advancements in machine learning, we replace coarse-grained matching with more precise deep learning approaches. Introducing the AI Impact measure, we employ Deep Learning Natural Language Processing to automatically identify AI patents that may impact various occupational tasks at scale. Our methodology relies on a comprehensive dataset of 17,879 task descriptions and quantifies AI’s potential impact through analysis of 24,758 AI patents filed with the United States Patent and Trademark Office between 2015 and 2022. Our results reveal that some occupations will potentially be impacted, and that impact is intricately linked to specific skills. These include not only routine tasks (codified as a series of steps), as previously thought but also nonroutine ones (e.g. diagnosing health conditions, programming computers, and tracking flight routes). However, AI’s impact on labor is limited by the fact that some of the occupations affected are augmented rather than replaced (e.g. neurologists, software engineers, air traffic controllers), and the sectors affected are experiencing labor shortages (e.g. IT, Healthcare, Transport).

**Significance Statement**

We introduce the Artificial Intelligence Impact (AII) measure*, utilizing Deep Learning Natural Language Processing to automatically identify AI patents affecting occupational tasks. Our findings reveal that:

1. AI’s impact on occupations defies simple categorizations of task routineness. It intricately affects specific skills within tasks, from routine (e.g. scanning items) to nonroutine (e.g. decision-making under stress by air traffic controllers), challenging the assumption that only routine tasks are susceptible.
2. AI’s impact on labor may be limited by the fact that some of the affected occupations are augmented rather than replaced, and some of the sectors affected are experiencing labor shortages.

* Project page: https://social-dynamics.net/aii

## Introduction

The rapid advancement of Artificial Intelligence (AI) has undeniably created new business opportunities (1) but has also reshaped the labor market (2–5), simultaneously reducing hiring in non-AI positions and altering the skill requirements of remaining job postings (6). Exemplifying this phenomenon, the manufacturing sector has witnessed the automation of previously human-intensive assembly line tasks, while chatbots and virtual assistants have taken over routine inquiries and support functions in customer services (7). Recent AI advances, including generative AI, may further (re)shape occupations over the long term, fueling growth in certain sectors and eroding others (8). AI automation has not only streamlined processes but has also generated economic benefits (9), enabling companies to allocate resources more effectively and to redirect human capital towards higher-value, creative, and complex tasks—*(re)skilling and upskilling* their workforce (10). However, this transformation has given rise to divergent viewpoints, with some scholars arguing for a future characterized by AI occupation displacement and mass unemployment (11, 12), while others posit that the AI revolution has the potential to enhance both productivity and quality of work (13).

Previous literature on AI impact on occupations has primarily focused on two classes of methodologies. The first measures the impact of AI on occupations. More specifically, it breaks down occupations into a finite set of abilities (e.g. manual dexterity, persuasion) and measures the impact on those abilities. Two examples that illustrate this approach are the Frey and Osborne’s (14) method and the AI Occupational Exposure (AIOE) method (3). Frey and Osborne’s method uses nine abilities extracted from O*NET database, covering 70 occupations that were manually labeled and extended with a classifier to 702, including roles like clergy, dentists, and chief executives. In contrast, AIOE uses 52 abilities derived from the O*NET database, focusing on 10 Electronic Frontier Foundation (EFF) applications such as image and speech recognition, and language modeling. However, both of these methods share a common limitation. Their reliance on coarse-grained abilities in the computation of AI’s impact may not fully capture the nuances of AI. Consider, for example, the ability of information ordering. Methods based on abilities may categorize tasks that involve organizing information in the same way, without distinguishing between the highly structured and complex information ordering required for database design and the simpler, routine information tasks of librarians such as alphabetizing files (15).

The second class of methodologies measures the impact of AI on tasks rather than occupations. This concept is illustrated by Brynjolfsson *et al.*’s Suitability for Machine Learning (SML) method (2), which measures the impact of AI using a comprehensive set of 18,156 tasks spanning 964 different occupations. However, SML relies on the assessments of crowdworkers to determine the suitability of specific tasks for machine learning. This reliance may introduce subjective biases from annotators (e.g. varying levels of expertise or cultural factors may lead to inconsistencies), and poses challenges in terms of scalability. Also, similar to Frey and Osborne’s and AIOE, SML is limited by the static nature of its one-time manual labeling. As technology advances and new capabilities emerge such as Large Language Models (LLMs), relying solely on a fixed set of abilities or subjective assessments of task suitability for automation becomes increasingly inadequate. For example, a copywriter is likely to be impacted by LLMs (16). However, if one were to examine copywriters at different points in time, such as in 2010 or 2015, the impact of AI on them would not be constant but would drastically change since language models were not as powerful back in 2010 as they are today. To fully capture the impact of a fast moving technology such as AI, therefore, it is crucial for methods to be adaptable to the ever-evolving technological advancements. To gauge the likelihood of automation, it is essential to identify which systems are poised for construction and commercialization. Annual business surveys serve as a source for measuring the adoption of automation. However, they may be subject to biases (e.g. respondents may over-report positive aspects, prioritize certain business operations, potentially neglect others, and interpret survey questions subjectively without standardized criteria) and are infrequently updated (17). An alternative, more objective source is patents. Patents are a typical source in scholarly work to identify emerging technological innovations (18–20). Prior work used patents to study the effect of automation and employment changes (21, 22). By analyzing the text of US patents granted between 1976 and 2014, Mann *et al.* (22) showed that the effect of automation differs across sectors. For example, the manufacturing industry, where most robots are used, experienced employment losses, while the service sector experienced employment gains; a finding that aligns with those reported by Autor and Dorn (23). More broadly, patents provide insights into emerging systems and technologies (24), leading Webb to study AI innovations by comparing occupational task descriptions with patent titles (5). This method employs a dictionary approach to identify verb–noun pairs associated with both tasks and patent titles. Another method, similar in its approach to Webb’s term matching, employs a normalized term matching approach to determine the similarity between tasks and patents (25), and does so in the specific area of robotics rather than AI. The method most similar to ours was proposed in (26, 27), in which, using word embeddings, patents are matched with broad occupation categories from the American Community Survey. However, that level of categorization is not suitable for researching the characteristics of specific jobs. Overall, this second class of approaches has used either term matching or word embedding. The problem is that term matching does not capture the semantic meaning of words (e.g. it does not distinguish between “bank” as in “data bank” or as in a financial institution) (28), and word embedding does not account for word ordering (e.g. “data entry and analysis” and “analysis of entry data” are considered similar based on word embeddings yet are two different tasks). As a result, these methods either miss relevant patents or return spurious task–patent matches, as detailed in Tables S5 and S6.

**Fig. 1.**
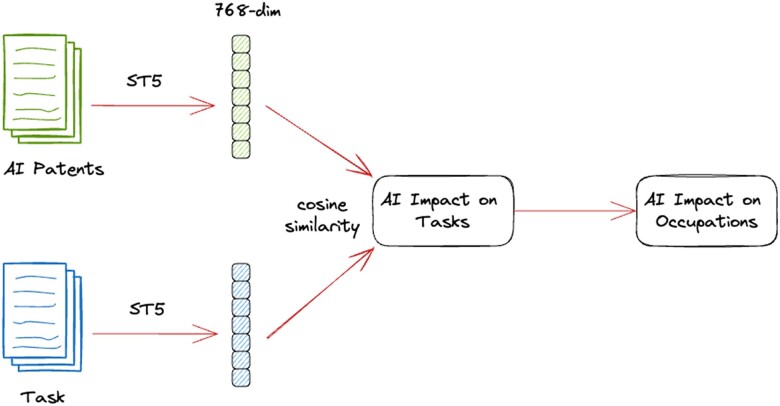
Framework automating AII measure. Using the Sentence-T5 (ST5) model, we first generated two vector representations (embeddings): one capturing the semantic meaning of an AI patent, and the other the semantic meaning of a task description. We then computed the patent–task cosine similarity from the embeddings on all patent–task pairs. This process was conducted to identify which tasks were impacted by which AI patents. Finally, for each occupation, we calculated the proportion of impacted tasks out of the total ones, and this proportion determined the AII of that occupation.

To overcome these limitations, we introduced and validated the AI Impact (AII) measure. AII utilizes 19,259 task descriptions from O*NET and assesses AI’s potential impact through innovations found in 24,758 AI patents filed with the United States Patent and Trademark Office (USPTO) from 2015 to 2022. Built on Sentence-T5 (ST5)(29), a natural language processing-focused deep learning framework (Fig. 1), the method gauges semantic similarity between occupation task descriptions and patent descriptions (explained in “Datasets”) by embedding not individual words but the entire document (e.g. the entire patent’s abstract), allowing for considering both semantic meaning and word ordering. The AII score is calculated in three steps. Firstly, the method identifies the most similar patent for each task based on maximum cosine similarity. Secondly, it categorizes a task as AI-impacted if its similarity with the most similar patent surpasses a threshold at the 90th percentile, as previous literature suggested (24) and this work further empirically validated (see “Task–Patent Matching” in Supplementary Material). We select, for each task, the closest patent rather than counting the number of closely linked patents. This approach provides a more targeted understanding of the specific innovations or solutions directly relevant to that particular task, avoiding dilution of the analysis with potentially less pertinent or peripheral patents. Also, given that patents can be general, our approach addresses this by using the text not only in titles, as previous approaches did (5), but also in abstracts, which are more likely to contain application domains or references to specific tasks (illustrated in Table S4). Finally, the AII score for an occupation is computed by dividing the number of tasks impacted by AI patents by the total tasks for that occupation (as detailed in “Measuring AI Impact (AII) on Occupation Tasks”). For insights into AI’s economic ramifications, the method aggregates AII scores for each occupation at industry sector-level.

## Results

### Validation with historically impacted occupations

To begin to understand the nature of the AII score, we first validated it empirically through two historical case studies: robots and software. We chose them for three reasons. First, their introduction into the labor market has been associated with reductions in employment and wages (30, 31). Second, due to the recent emergence of these technologies, they are likely to provide insights into how the economy may respond to the introduction of AI. Lastly, these two historical cases have been used in previous works to empirically assess methodologies similar to ours (5), offering a basis for comparison.

We therefore adjusted the AII score to encompass exposure to robots and software rather than AI (as detailed in “Impact of Robots and Software” of Supplementary Material) by focusing on patents related to these two technologies. We studied how robot and software exposure affected employment and wages using US census data from 1980 to 2010. Following Webb’s methodology (5), which controlled for industry effects, educational levels, wage polarization, and off-shorability, we found that introducing robots led to a 9% decrease in employment and a 4% decrease in wages, and that introducing software resulted in a 10% decrease in employment and a 7% decrease in wages during this period. These findings align with Webb’s, indicating that occupations exposed to robots or software have decreased in number and pay lower wages. However, Webb’s method, which relies on keyword matching, sometimes includes patents that should not be matched with certain job tasks (as detailed in “Previous Attempts to Link Tasks to Patents” in Supplementary Material), leading to larger decreases and an overestimation of AI’s potential impact compared to our estimates.

### Most- and least-impacted occupations

We compared the potentially most-impacted (highest AII scores) occupations with the least-impacted (lowest AII scores) occupations (Table 1 only reports the 20 most- and least-impacted occupations for brevity and comparability with previous methods), and did so by thematically analyzing the AI patents associated with the tasks of each group’s occupations (as described in “Thematic Analysis on Occupations and Industry Sectors”).

**Table 1.** 20 most- and least-impacted occupations ranked by the Artificial Intelligence Impact (AII) measure.

Rank	Most-impacted	Least-impacted
1	Cardiovascular Technologists and Technicians	Pile Driver Operators
2	Sound Engineering Technicians	Dredge Operators
3	Nuclear Medicine Technologists	Aircraft Cargo Handling Supervisors
4	Air Traffic Controllers	Graders and Sorters, Agricultural Products
5	Magnetic Resonance Imaging Technologists	Insurance Underwriters
6	Electro-Mechanical and Mechatronics Technologists and Technicians	Floor Sanders and Finishers
7	Orthodontists	Reinforcing Iron and Rebar Workers
8	Power Distributors and Dispatchers	Farm Labor Contractors
9	Neurologists	Administrative Services Managers
10	Industrial Truck and Tractor Operators	Rock Splitters, Quarry
11	Public Safety Telecommunicators	Brokerage Clerks
12	Computer Numerically Controlled Tool Programmers	Podiatrists
13	Security Guards	Helpers–Painters, Paperhangers, Plasterers, and Stucco Masons
14	Remote Sensing Scientists and Technologists	Shipping, Receiving, and Inventory Clerks
15	Machinists	Cooks, Short Order
16	Radiologists	Team Assemblers
17	Atmospheric and Space Scientists	Proofreaders and Copy Markers
18	Computer Numerically Controlled Tool Operators	Butchers and Meat Cutters
19	Textile Knitting and Weaving Machine Setters, Operators, and Tenders	Door-to-Door Sales Workers, News and Street Vendors, and Related Workers
20	Medical Transcriptionists	Segmental Pavers

#### Most-impacted occupations

**Fig. 2.**
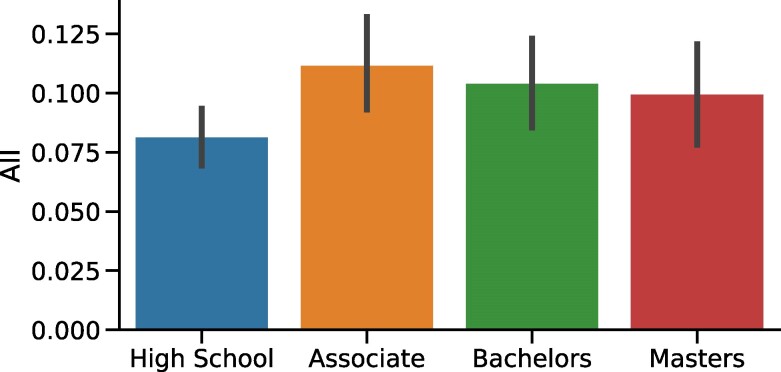
AII score binned by the level of education: high school, associate degrees from community colleges, bachelor’s degrees, and master’s degrees in science. This binned score was obtained by averaging the scores across all occupations in a given education category, weighted by the total employment for those binned occupations.

The highest-impact occupations mainly consist of white-collar occupations such as cardiovascular technicians, sound engineers, nuclear medicine technologists, air traffic controllers, and magnetic resonance imaging technologists. Indeed, the AII score, binned by education levels from high school to Master of Sciences, shows that the highest impact is seen for jobs requiring degrees from community colleges or Bachelor’s degrees (Fig. 2) rather than high school diplomas or lower qualifications. Highest-impacted occupations are primarily found in the healthcare, information technology, and manufacturing. Their tasks can be completed in a very specific sequence, and the inputs and outputs of these tasks can be expressed in a machine-readable format. To see how, we examined the types of tasks that patents have automated, and organized the patents into three themes: *healthcare*, *information technology*, and *manufacturing* (Table 2). For the theme of healthcare, from 2015 to 2022, 60% of the tasks done by cardiovascular technologists and 48% of those done by magnetic resonance imaging (MRI) technologists have been impacted by patents automating health records’ management and analyzing MRI scans. In addition to the advanced healthcare occupations, we observed a significant number of patents impacting less skilled healthcare personnel including, for example, patents recording and evaluating patient questionnaires. For the theme of information technology, over the same five years, 47% of tasks done by software developers and 40% of those done by computer programmers have been impacted by patents automating programming tasks and developing workflows. For the theme of manufacturing, over the same 5 years, 45% of tasks done by truck and tractor operators and 40% of earth drillers’ tasks have been automated. These automated tasks are planning processes such as water-well drilling rigs and driving through electric-powered trucks.

**Table 2.** The most-impacted occupations based on AII scores.

Rank	Occupation	Sector	Task	Patent	Similarity	# Impacted Tasks	Total Tasks	AII
1	Cardiovascular Technologists and Technicians	Healthcare	Observe gauges, recorder, and video screens [⋯] during imaging of cardiovascular system.	Automated analysis of vasculature in coronary angiograms	0.8753	16	25	0.64
2	Sound Engineering Technicians	Arts and entertainment	Record speech, music, and other sounds on recording media, using recording equipment.	Media capture and process system	0.8293	8	14	0.57
3	Nuclear Medicine Technologists	Healthcare	Process cardiac function studies, using computer.	Electrocardiogram analysis	0.8645	9	17	0.53
4	Air Traffic Controllers	Transportation	Determine the timing or procedures for flight vector changes.	Constraint processing as an alternative to flight management systems	0.8298	12	23	0.52
5	Magnetic Resonance Imaging Technologists	Healthcare	Operate optical systems to capture dynamic magnetic resonance imaging (MRI) images [⋯]	MRI system and method using neural network for detection of patient motion	0.8615	12	23	0.52
6	Electro-Mechanical and Mechatronics Technologists and Technicians	Manufacturing	Train robots, using artificial intelligence software or interactive training techniques [⋯]	Backup control based continuous training of robots	0.8846	18	35	0.51
7	Orthodontists	Healthcare	Study diagnostic records, such as medical or dental histories [⋯] to develop patient treatment plans.	Patient-Specific Therapy Planning Support Using Patient Matching	0.8460	5	10	0.50
8	Power Distributors and Dispatchers	Utilities	Control, monitor, or operate equipment that regulates or distributes electricity or steam [⋯]	Power grid aware machine learning device	0.8562	7	15	0.47
9	Neurologists	Healthcare	Interpret the results of neuroimaging studies, such as [⋯] Positron Emission Tomography (PET) scans.	Pet quantitative localization system and operation method thereof	0.8352	11	24	0.46
10	Industrial Truck and Tractor Operators	Manufacturing	Move controls to drive gasoline- or electric-powered trucks, [⋯]	Autonomous Truck Unloading for Mining and Construction Applications	0.8301	5	11	0.45
11	Public Safety Telecommunicators	Public administration	Test and adjust communication and alarm systems, and report malfunctions to maintenance units.	Security-Relevant Diagnostic Messages	0.8237	8	18	0.44
12	Computer Numerically Controlled Tool Programmers	Manufacturing	Determine the sequence of machine operations, and select the proper cutting tools [⋯]	Methods and apparatuses for cutter path planning and for workpiece machining	0.8376	7	16	0.44
13	Security Guards	Administrative & support services	Operate detecting devices to screen individuals and prevent passage of prohibited articles into restricted areas.	Touchless, automated and remote premise entry systems and methods	0.8566	6	14	0.43
14	Remote Sensing Scientists and Technologists	Manufacturing	Develop automated routines to correct for the presence of image distorting artifacts, such as ground vegetation.	Method for plantation treatment based on image recognition	0.8413	10	24	0.42
15	Machinists	Manufacturing	Machine parts to specifications, using machine tools, such as lathes, milling machines, shapers, or grinders.	Machining equipment system and manufacturing system	0.8440	12	29	0.41
16	Radiologists	Healthcare	Perform or interpret the outcomes of diagnostic imaging procedures including magnetic resonance imaging (MRI), computed tomography (CT), positron emission tomography (PET), [⋯]	Systems and methods for integrating tomographic image reconstruction and radiomics using neural networks	0.8569	7	17	0.41
17	Computer Numerically Controlled Tool Operators	Manufacturing	Implement changes to machine programs, and enter new specifications, using computers.	Registering collaborative configuration changes of a network element in a blockchain ledger	0.8370	11	27	0.41
18	Atmospheric and Space Scientists	Scientific and technical services	Analyze historical climate information, such as precipitation or temperature records, to help predict future weather or climate trends.	Combining forecasts of varying spatial and temporal resolution	0.8429	11	27	0.41
19	Textile Knitting and Weaving Machine Setters, Operators, and Tenders	Manufacturing	Set up, or set up and operate textile machines that perform textile processing [⋯]	Parameter Manager, Central Device and Method of Adapting Operational Parameters in a Textile Machine	0.8477	8	20	0.40
20	Medical Transcriptionists	Healthcare	Transcribe dictation for a variety of medical reports [⋯]	Methods for improving natural language processing with enhanced automated screening for automated generation of a clinical summarization report and devices thereof	0.8367	6	15	0.40

Each occupation’s score is calculated as the number of impacted tasks divided by the total number of tasks for that occupation. For each occupation, a task–patent pair is presented, corresponding to the most-impacted task by the patent (i.e. the patent with the highest similarity score to the task) which is determined by calculating the textual similarity between the task’s description and a patent’s title plus abstract.

Studies on long-haul truck driving show that truckers are not being replaced by AI (32). Instead, they are now using smart technology that monitors their health, like smartwatches and advanced health devices. Previous research did not account for these new technologies that came out over the years (3). Our score, on the other hand, is adjusted based on new innovations, so it changes over time.

**Fig. 3.**
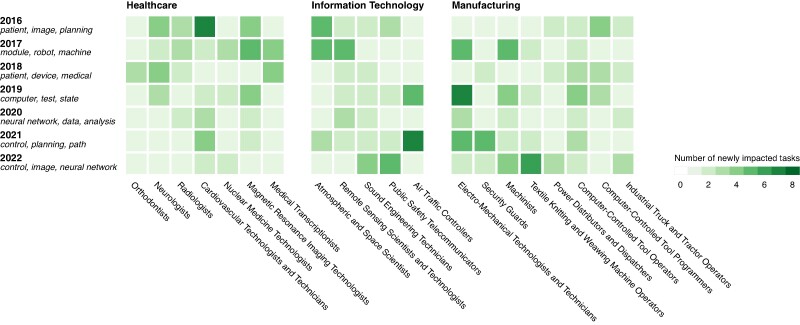
The number of newly impacted tasks each year for the most affected occupations, combined with the most frequently occurring words in the patents influencing those tasks, are organized around the themes of healthcare, information technology, and manufacturing. These were derived qualitatively and describe the main themes emerging from the patents. Between 2016 and 2018, patents mentioned “patient”, “image”, “planning”, “medical”, “device” matched tasks in healthcare. Between 2019 and 2021, patents mentioned “data”, “analysis”, and “neural networks” matched tasks in information technology. Between 2021 and 2022, patents mentioned “control”, “planning”, “path”, “user”, “image”, and “neural network” matched tasks in manufacturing.

By analyzing the annual increase in impacted tasks and examining the most frequently occurring words in patents’ abstracts each year (Fig. 3), we identified two categories of highly impacted occupations. The first category includes occupations that experienced a sudden impact, while the second category includes occupations that faced continuous impact over time.

Occupations that underwent sudden impacts are predominantly within the healthcare sector. This impact became most pronounced in 2016, when eight new tasks were affected, gradually decreasing to just one new impacted task by 2019. In 2016, patents began to significantly impact healthcare by automating medical imaging and diagnosis through machine learning models, devising treatment plans and medical devices, and recording and analyzing patient data. This impact continued into 2017 and 2018, although to a lesser degree, focusing on predicting optimal radiation therapy doses, dental treatment plans, and processing medical patient data.

Occupations that sustained continuous impact over time are primarily in information technology and manufacturing. In the information technology sector, occupations such as software developers, and in manufacturing, occupations such as earth drillers, saw consistent increases from zero new impacted tasks in 2016 to six new impacted tasks in 2019. In information technology, the potential impact of patents became noticeable in 2017 when they began training robots to design and execute iterative tests on chemical samples, working on aerial and satellite imagery to create products such as land cover maps, and implementing speech recognition and natural language processing on audio. This impact steadily rose and extended into 2022, with patents integrating machine learning into software systems, automating tasks such as troubleshooting networks and code reviews. In manufacturing, patent potential impact emerged in 2018, focusing on optimizing supply chain logistics and planning material dumping operations. This impact persisted into 2022, further supporting predictive maintenance and operational optimization such as determining aircraft conditions, with patents integrating reinforcement learning and other advanced neural networks.

#### Least-impacted occupations

The least-impacted occupations mainly consist of blue-collar occupations such as pile-driver operators, dredge operators, aircraft cargo handling supervisors, agricultural graders and sorters, and insurance underwriters. Again, the AII score, binned by education levels, shows that the lowest impact is seen for jobs requiring a high school diploma or less (Fig. 2). These occupations are primarily found in agriculture, transportation, accommodation and food services, and construction sectors, where the core tasks and responsibilities revolve around physical and manual labor and typically do not require a wide range of complex mental or physical activities, nor do they involve abstract reasoning. In agriculture, least-impacted tasks involve food inspection and dairy management for livestock. In transportation, such tasks involve scheduling and resource allocation for airline operations, engine sound control, and vehicle dispatching. In accommodation and food services, tasks include monitoring and recording food temperatures. In construction, such tasks involve the maintenance and operation of equipment and machinery. In addition to these sectors, by examining occupations with nearly zero AII scores, we found another least-impacted set of occupations: managerial ones. Contrary to the previous least-impacted occupations which involve manual labor or dexterity, managerial occupations typically require human interactions, and scarce expert knowledge tacitly acquired over years of experience, having tasks ranging from contract negotiation to proposal review to internal assessments and audits. Overall, AI’s impact on occupations is limited because: (i) some occupations are augmented by AI rather than replaced and (ii) the industries being affected already have a shortage of workers.

### Augmented rather than replaced occupations

**Fig. 4.**
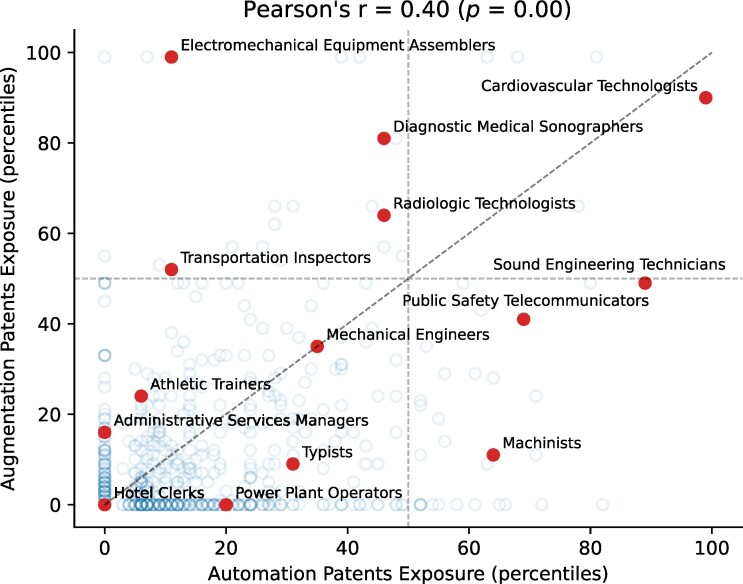
Automation vs. augmentation using patent similarity to tasks and micro-titles defined in the Census Alphabetical Index of Occupations and Industries (CAI) (26).

Our AII captures AI’s potential for automation. However, previous work has differentiated between automation and augmentation. To account for that, we implemented Autor *et al.*’s method (26) to compute AI’s potential for augmentation (explained in “Materials and methods”). We found that certain occupations will not be replaced by AI, but instead will be augmented by it (top left quadrant in Fig. 4). For example, the role of a hearing aid specialist involves a significant human element, especially in understanding and responding to patients’ emotional, psychological, and physical needs. This is reflected in the lack of patents that match the task of “counseling patients and families on communication strategies and the effects of hearing loss”; a task that requires empathy and emotional intelligence, which is likely no current AI innovation can automate. Another example is that of electrical and electronics repairers, which relies heavily on technical skills, detailed knowledge, and hands-on interaction. Consider that occupation’s task of “consulting with customers, supervisors, or engineers to plan the layout of equipment or to resolve problems in system operation or maintenance”. While automation can assist in some aspects, human expertise and decision-making are crucial. This task has not been matched with any patent. In general, for occupations that may be augmented by AI, AI will advise, coach, and alert decision-makers as they apply expert judgment.

### Affected sectors experiencing labor shortages

**Fig. 5.**
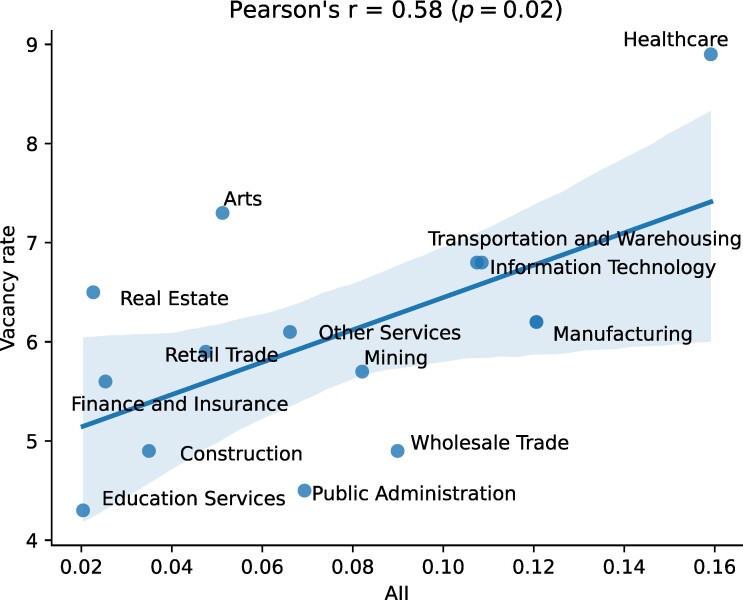
Job vacancy rates by sector vs. sector-level AII. The sector of Accommodation and Food Services was positioned more than two standard deviations away from the regression line (i.e. considered as an outlier) and was removed. The original plot is in Supplementary material.

To ascertain whether AI patents are linked to labor supply constraints or to labor demand in a sector, we correlated the AII industry sector scores with the annual vacancy rates from each sector in 2022. We found that AII and vacancy rates are positively correlated. After removing the outlier sector of Accommodation and Food Services—positioned more than two standard deviations away from the regression line (Fig. S7)—the correlation was positive and stronger, with a Pearson’s correlation coefficient of r=0.58 with p=0.02 (Fig. 5). This suggests that sectors potentially impacted by AI are currently experiencing labor shortages.

**Fig. 6.**
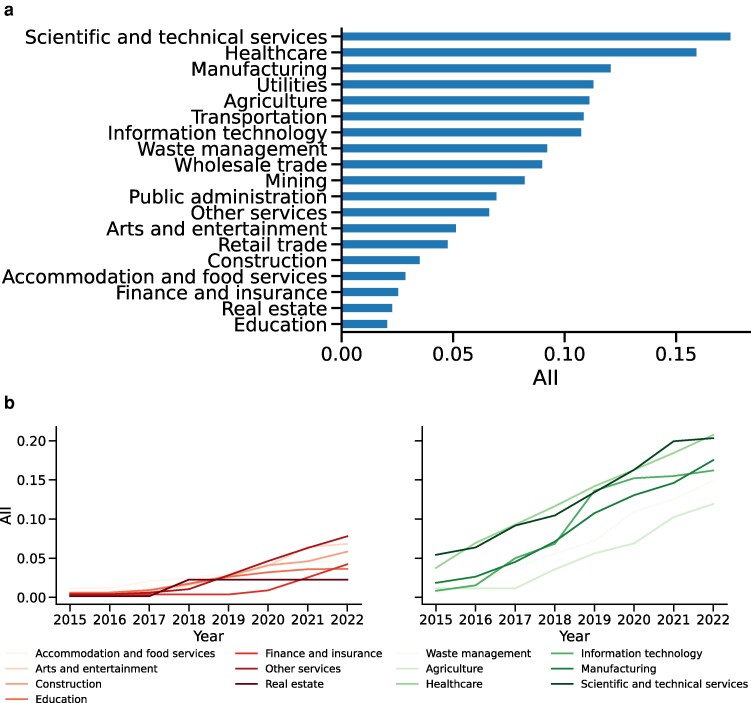
Sector-level AII scores for: a) all sectors; b, left) sectors with lowest rate of change from 2015 to 2022; and b, right) sectors with highest rate of change.

Highly impacted sectors include healthcare, information technology, and manufacturing, which aligns with our thematic analysis of tasks within the most-impacted occupations (Fig. 6). These sectors have experienced a significantly high rate of impact. From the thematic analysis, two possible explanations emerge. The first explanation lies in the nature of the tasks within these sectors, which are likely to be replaced by AI-enhanced hardware. For example, in healthcare, tasks involving the use of X-rays or MRI scans, such as those used by radiologists, have been automated by patents on advanced medical equipment and devices. Similarly, in manufacturing, tasks that involve the examination of chemical or biological samples, such as those performed by food science technicians, can now be executed by AI-enhanced hardware. The second explanation is that the occupations within these sectors entail tasks demanding extensive data analysis and processing. In information technology, film editors, for example, engage in video data editing, which our patent analysis found to have been streamlined by AI-based software. Likewise, scientists in healthcare, information technology, or manufacturing often handle large volumes of data, and recent patents deal with both structured and unstructured data (e.g. using deep-learning for computational biology (33)).

## Discussion

### Consensus and discrepancies in the literature

Unlike previous methods for assessing the potential impact of AI on occupations, which either rely on a finite set of abilities (e.g. manual dexterity) linked to specific occupations (3, 14) or employ subjective evaluations to determine tasks’ suitability for automation (2), our AII measure provides an objective approach by leveraging patent data to capture the dynamic landscape of technological advancements in AI.

To position our findings, we explore the consensus or lack thereof of which occupations will be potentially impacted by AI in the literature.

#### Consistent results in the literature

Frey and Osborne’s (14), AIOE (3), SML (2), and Webb’s (5) concur that low-skilled occupations typically involving human labor are not impacted by AI. These occupations are typically found in industry sectors such as construction, where tasks primarily involve manual labor and the operation of machinery. Our AII measure aligns with these observations by categorizing such occupations as among the least impacted by AI. Least-impacted sectors include construction, accommodation and food services, real estate, education, public administration, and finance and insurance. These sectors have experienced a significantly low rate of impact. From the thematic analysis, three possible explanations emerge. The first explanation centers on the fact that occupations within certain sectors are often associated with low-skilled or physical/manual labor. For instance, in construction, tasks range from assembling solar panels to maintaining pipe systems to operating various drills, all of which require physical labor. Additionally, manual dexterity is challenging to automate. In public administration, numerous occupations, such as police officers and firefighters, still require manual skills. The second explanation is that occupations within certain sectors demand basic and nonspecialized skills. For instance, in accommodation services, the tasks of waiters and baristas typically involve minimal specialized knowledge or vocational training, such as serving food and drinks. Similarly, in real estate, brokers or clerks primarily require training in overseeing transactions and handling tasks related to office operations.The third explanation is that occupations within certain sectors often require a high level of skill and involve extensive interpersonal interactions. For instance, in managerial positions (e.g. CEOs), tasks typically encompass responsibilities such as delegating tasks, attending events and meetings, and negotiating contracts—all of which heavily rely on human interpersonal communication. Similarly, in education, a teacher’s role primarily revolves around delivering educational materials in person, a task that demands both physical presence and a higher level of education and specialized knowledge. In the legal sector, potentially impacted occupations include those involved in drafting legal documents or transcribing pretrial and trial proceedings, such as court reporters, in alignment with previous qualitative analyses on legal occupations (34). Conversely, roles requiring the design of bespoke legal solutions remain unaffected. Notably, client-facing tasks in the legal sector are also not impacted, resulting in an overall expectation of limited impact on the legal sector. Finally, in the financial sector, there are still occupations that necessitate human interactions, such as clerks, sales agents, and tellers.

#### Inconsistent results in the literature

While there has been a clear consensus in previous literature regarding manual labor occupations, we have identified four types of occupations for which consensus has been so far lacking. These categories include: occupations requiring basic and nonspecialized skills; occupations requiring high skills and interpersonal interaction-based occupations; those where tasks are replaced by AI-enhanced hardware; and those involving extensive data analysis and processing. *Occupations that require basic and nonspecialized skills (least impacted according to our approach).* Frey and Osborne’s findings (14) indicated that occupations within the accommodation and food services sector (e.g. cooks, dishwashers, waiters, bartenders) are highly impacted. Similarly, Webb’s findings suggest that nonroutine manual occupations are highly impacted; there are patents matching those occupations, which may well be coarse-grained matches, as exemplified in Table S5. However, SML (2) and AIOE (3) found the very same occupations to be among the least-impacted. Our approach aligns with SML and AIOE, identifying these occupations as among the least-impacted due to the absence of AI patents automating the manual tasks associated with them. *Occupations that are highly skilled and involve interpersonal interactions (least impacted according to our approach).* AIOE (3) found that occupations requiring high skills and interpersonal interactions, such as those in the education sector (e.g. teachers) or managerial positions (e.g. CEOs), are highly impacted. Similarly, Webb found that occupations that require interpersonal tasks are hard to automate (5). In contrast, SML (2) found these occupations, particularly managerial positions, to be among the least impacted, aligning with Frey and Osborne’s method (14). However, in the case of SML, there was a distinction within occupations involving interpersonal interactions. While SML found managerial positions to be among the least impacted, it identified occupations such as concierges, which also involve interpersonal interactions, as more likely to be impacted by AI. Upon closer examination of the SML method, which relies on rubrics (i.e. a type of scoring guide for crowdworkers to assess the suitability of tasks for machine learning, as shown in Table S2), we noted that the corresponding definition in the rubrics entailed a “wide range of interactions,” making it challenging to capture their nuances. Additionally, since SML relies on crowdsourced data, there is a potential for subjective bias or a lack of full understanding of the nuances of interpersonal interactions. Finally, interactions were captured by only one item out of the 23 items in the rubrics making that item’s contribution to the overall score limited. Our approach aligns with Frey and Osborne’s (14), Webb’s (5) and, to some extent with SML (2) as no AI patents were found to target these occupations. *Occupations that consist of tasks that are replaced by AI-enhanced hardware (most impacted according to our approach).* Frey and Osborne (14) discovered that occupations in healthcare, such as MRI and cardiovascular technologists, which are likely to be replaced by AI-enhanced hardware, were among the least-impacted. In contrast, SML (2) and AIOE (3) identified these same healthcare occupations as among the most-impacted ones. Webb (5) found that occupations involving nonroutine manual tasks (e.g. operating devices or equipment in healthcare or manufacturing) to be among the most-impacted ones. Our approach aligns with SML, AIOE, and Webb’s method due to the presence of AI patents automating tasks using AI-enhanced hardware. For example, in healthcare, patents have automated tasks such as medical imaging and diagnosis using machine learning models, the development of treatment plans, the creation of medical devices, the recording and analysis of patient data, and even the prediction of optimal radiation therapy doses, dental treatment plans, and the processing of medical data. *Occupations that require extensive data analysis and processing (most impacted according to our approach).* Frey and Osborne (14) found that occupations that require extensive data analysis and processing, such as those in information technology or manufacturing (e.g. software developers, chemists, aerospace engineers), were classified as among the least-impacted. In contrast, SML (2) and AIOE (3) categorized these very same occupations as among the most-impacted. Similarly, Webb (5) identified that occupations involving nonroutine cognitive analytic tasks to be among the most-impacted ones. Our findings align with SML, AIOE, and Webb’s method due to the presence of patents related to tasks that require data analysis and processing. For example, patents about training robots for task execution, image and video processing, speech recognition, and natural language processing, as well as the integration of machine learning into software systems. These patents automate tasks such as troubleshooting networks, code reviews, optimizing supply chain logistics, planning material dumping operations, and supporting maintenance, including determining aircraft conditions. Our findings are further confirmed by recent works studying the potential exposure of occupations and tasks to Generative AI (35, 36). They found that Generative AI will potentially impact high-skilled, intellectual, or creative professions (e.g. mathematicians, writers, and translators), where these models can effectively augment capabilities in data analysis, writing, and language translation.

### Implications

The impact of AI on occupations carries important implications for the workforce. However, when placed our findings within the context of previous literature, it became evident that there is a lack of consensus regarding which occupations will be affected and which will remain unaffected. As an initial step, we contend that achieving consensus is crucial to start formulating effective policies to address the ongoing transformations in the labor market. To initiate this process, with our findings in mind, we outline three key areas in which initiatives can be developed.

#### Initiatives for white-collar workers

Policymakers and employers should launch specific initiatives targeting white-collar occupations in sectors such as information technology, manufacturing, and healthcare. These initiatives can equip workers with the skills needed for high-value, creative, and complex tasks. For example, a manufacturing worker can undergo training in robotics programming, enabling them to effectively operate and maintain AI-driven machinery. Similarly, healthcare professionals can acquire telemedicine and data analytics skills to enhance patient care and diagnostics.

#### Initiatives for blue-collar workers

Blue-collar occupations, predominantly found in agriculture, accommodation and food services, and construction, typically involve low-skilled work demanding physical labor. While previous literature has suggested reallocating low-skilled workers to tasks less susceptible to computerization (14), such as those requiring creative and social intelligence, we argue that (re)skilling and upskilling (10) should be approached cautiously. That is because regions that excessively rely on knowledge-based economies are likely to face significant AI impact.

#### Initiatives for continuous learning and interdisciplinary training

Promoting a culture of continuous learning and skill development is essential. Employers can encourage individuals to embrace lifelong learning through online courses, certifications, and vocational training, enabling them to adapt to the changing occupational landscape (37). Similarly, encouraging interdisciplinary training can prepare the workforce for the demands of AI-augmented occupations. For example, blending traditional engineering with AI and machine learning training can create a workforce capable of developing and maintaining AI-enhanced systems across sectors.

### Limitations and future work

This work has five primary limitations. First, our analysis is conducted on an annual basis and assumes that the tasks associated with a given occupation in the O*NET database remain consistent across all versions within the same year.

Second, our method relies on patent abstracts to provide a finer-grained understanding of occupational tasks, mainly from the US-focused USPTO dataset. Despite the most significant innovations are typically patented in all major patent jurisdictions (22), the US holds a distinct position. In 2014, nearly a quarter of the approximately 10.9 million patents worldwide were granted in the United States, highlighting its significant share (38). However, more recently, Carbonero *et al.* (39) and Guarascio *et al.* (40) studied the potential impact of AI on occupations in Southeast Asia and Europe. Unlike our method, their methods relied on manual annotations to determine the suitability of specific tasks for machine learning. We therefore calculated the AII score based on US patents alone and compared it with the AII score from patents combined from the United States, China, Japan, and South Korea, finding them to be highly correlated (r=0.93), leaving our results unaffected. However, future research should still replicate our method and explore potential cultural differences in how patents are written and used in other contexts, not least in the European Union.

Third, it is important to acknowledge that the existing portfolio of USPTO patents may not comprehensively cover all the innovations that may impact a particular occupation. To address this limitation, one potential approach could involve supplementing patent data with other sources, including research papers and code repositories (41).

Fourth, our assumption is that the likelihood of an AI system being built is determined by whether it is patented. While generally true, there are exceptions. A patented system may not be built, as the patent could be intended for defensive or offensive purposes (42, 43). Conversely, a nonpatented system might still be constructed, with its design protected by secrecy or trademarks (44, 45). While there are numerous patents for systems designed to improve meetings, calendaring, and instant messaging, patents focused on interpersonal interactions may be less common. However, even if these patents do not directly threaten jobs requiring human interaction, they could still have secondary effects. For example, if AI significantly affects occupations like artists or software developers, managerial positions may become less essential due to a reduced workforce needing oversight. Yet, our method does not capture such cascading impacts on the job market. Furthermore, patenting rates vary among sectors (46), and there is a lag between an innovation being patented and its use and impacts diffusing across the economy (41).

Finally, our analysis is based solely on a concise yet comprehensive 7-year time window, spanning from 2015 to 2022. We also repeated the analysis for 2010–2022 and found no significant difference in the results (as shown in Table S8 and Fig. S5). This does not capture emerging technologies, such as Large Language Models (LLMs). Future research could replicate our methodology to assess the potential impact of emerging technologies, such as cryptocurrency, the metaverse, and LLMs by using upcoming patents in those fields.

## Materials and methods

### Datasets

#### Occupation dataset

We collected detailed task descriptions for a wide range of occupations from the O*NET database (47), a widely used source in occupational studies (2, 3, 48–50). In total, we collected 759 unique occupations and 17,879 unique tasks from O*NET 26.3 version released in May 2022. The distribution of these tasks ranges from 4 to 286, with a median of 20 tasks per occupation (Fig. S1).

#### Patent dataset

To obtain a corpus of AI patents, we first retrieved 74,875 patents granted by the United States Patent and Trademark Office (USPTO) between 2015 and 2022 that were classified to be about AI based on an official way of coding patents called PATENTSCOPE AI Index (51) to then filter away patents only tangentially related to AI. We selected the subset of patents in the index class core AI applications (Table S1). This resulted in a final corpus of 24,758 AI patents.

### Measuring AII on occupation tasks

#### Sentence-transformers

We developed a Sentence-Transformers Deep Learning framework (52) for Natural Language Processing that uses the Sentence-T5 (ST5) architecture (29) to convert input text into “semantic vector representations” called “embeddings”. These embeddings capture the semantic information of the text and allow us to mathematically compute the similarity of a pair of text snippets. In particular, we chose the Sentence-T5-XL model, which is highly recommended for its effectiveness in handling various language tasks such as classification and similarity comparisons (52). This model has demonstrated exceptional performance in a comprehensive benchmark test—the Massive Text Embedding Benchmark—that evaluated different models across 58 datasets and 112 languages for embedding tasks such as classification, clustering, and semantic textual similarity (53). We used the default parameters of the model because they were already optimized for textual similarity tasks similar to ours. The model’s default training parameters include an Adafactor optimizer at a learning rate starting at 0.0001, with linear decay after 10% of the total training steps; the fine-tuning was conducted using a batch size of 2048, and a softmax temperature *τ* of 0.01 was used. The model was trained on a dataset of 2 billion question and answer (Q&A) pairs from online Q&A communities, and was then fine-tuned to enhance its understanding of how sentences are related to each other by training on pairs of sentences that had been manually reviewed for their meaning (54). The model uses a siamese network architecture (55), which processes pairs of sentences to generate a consistent output length, regardless of the sentence length. This method of producing fixed-size feature vector representations has proven effective in capturing the deeper meanings of text without the need for any preprocessing (56, 57).

#### AI impact

We defined AII as a measure of *“the exposure to AI by measuring the extent to which an occupation’s tasks are associated with patents”*. For each task, we identified the patent with the highest task–patent similarity score (Eq. 1) to represent the AI potential impact $\alpha_{t}$ on task *t* indicating the extent to which task *t* aligns with AI-related innovations:


(1) $$\alpha_{t}=\max_{p}\text{sim}(v_{t},v_{p}),$$

where $\text{sim}(v_{t},v_{p})$ is the cosine similarity between the embeddings of task *t* and the embeddings of patent *p*. We computed the impact of AI on task *t* by taking the maximum similarity value. We took the maximum instead of, say, the average because if the average was used, similarity scores from patents that are not particularly relevant to the task would be factored into the calculation, thereby diluting the AI impact score (Fig. S3). Multi-instance learning was also considered as an alternative, but it did not produce any more accurate task–patent matching (explained in “Task–Patent Matching” in the Supplementary material).

### Measures of AII on occupations and industry sectors

#### AI impact on occupations

We computed the AI impact $x_{j}$ on occupation *j* by computing the number of *AI-impacted tasks* over the total number of *j*’s tasks:


(2) $$x_{j}=\frac{\sum_{t\in \mathrm{tasks}(j)}1_{\alpha_{t}>p_{90}(\alpha )}}{\sum_{t\in \mathrm{tasks}(j)}1},$$

where $p_{90}(\alpha )$ is the 90th percentile of AI impact values computed on all occupations’ tasks, and $1_{\alpha_{t}>p_{90}(\alpha )}$ is an indicator function whose value is 1, if $\alpha_{t}>p_{90}(\alpha )$, and 0 otherwise. In other words, the AII measure is based on counting an occupation’s tasks impacted by AI without accounting for the relative importance of each task, in a way similar to previous work (26). Using the 90th percentile as the threshold makes the AI impact measure more robust to noise, which was also suggested in a previous study (24). Given that every task is assigned a similarity value in the previous step, the patent deemed most similar for a specific task might still be unrelated to that task. On the other hand, a higher 95th percentile threshold would be too strict, as 55% of the occupations would have zero impacted tasks. To further validate our task–patent matching method, two authors independently assessed the relevance of a patent to a task in a random sample of 100 task–patent pairs. Overall, their agreement was nearly perfect, with a Cohen’s Kappa of 0.84.

#### AI impact on industry sectors

To determine the potential impact of AI on industry sector *s*, we calculated the mean AII score across all occupations *j* associated with sector *s* (Eq. 3). Occupation *j* was assigned to sector *s*, if over 50% of workers in *j* were employed in *s*:


(3) $$\pi_{s}=\frac{1}{N_{s}}\sum_{\;j\in \mathrm{occupations}(s)}x_{j},$$

where $N_{s}$ is the number of occupations associated with *s*. If more than half of the workers in an occupation are employed in a particular sector, it can be reasonably concluded that this occupation is primarily associated with that sector (3). Lower thresholds might lead to occupations being associated with multiple sectors, making the results less specific. Conversely, a higher threshold might be too restrictive, potentially excluding occupations with a significant presence in a sector, even if not overwhelmingly so.

### Thematic analysis on occupations and industry sectors

#### Occupations

To identify emergent themes that characterize least- or most-impacted occupations, we conducted a thematic analysis (58, 59) on the task–patent pairs for all tasks associated with those occupations. This process consists of two steps: open coding, in which textual data is broken up into discrete parts and then coded; and axial coding, in which the researcher draws connections between the generated codes (60). We first applied open coding to identify key concepts that emerged across multiple task–patent pairs; specifically two of the authors read all task–patent pairs, and marked them with keywords that reflected the key concepts expressed in the text. They then used axial coding to identify relationships between the most frequent keywords to summarize them in semantically cohesive themes (e.g. healthcare, information technology, and manufacturing). Themes were reviewed in a recursive manner rather than linear, by re-evaluating and adjusting them as new task–patent pairs were parsed.

#### Industry sectors

Just as with occupations, we conducted thematic analysis (58–60) to uncover potential explanations for the most- and least-impacted sectors. We again applied open coding to identify key concepts that emerged on the descriptions of occupations (examples presented in Table S3) across the industry sectors under study, with the same two authors reading all descriptions and marking them with keywords that reflected the key concepts expressed in the text. We then again used axial coding to identify relationships and potential explanations.

### Beyond automation: measuring augmentation

Automation refers to “technologies that substitute for the labor inputs of occupations, potentially replacing workers performing these tasks” (26). That is what our AII captures, and it does so by first identifying the patent most similar to an occupation task (Eq. 1), and then computing the number of AI-impacted tasks over the total number of tasks at a given occupation (Eq. 2). In addition to automation, previous research introduced a complementary type of AI’s potential impact: augmentation. This refers to “technologies that increase the capabilities, quality, variety, or utility of the outputs of occupations, potentially generating new demands for worker expertise and specialization” (26). To measure augmentation, instead of measuring the similarity between patents and occupation tasks, we measured the similarity between patents and micro-titles defined in the Census Alphabetical Index of Occupations and Industries (CAI) (26), and then computed the number of AI-impacted micro-titles over the total number of micro-titles at a given occupation. Unlike occupation tasks, micro-titles capture the “emergence of new work categories that typically reflect the development of novel expertise within existing work activities (e.g. electrical trades skills specific to solar installations) or an increase in the market scale of a niche activity (e.g. nail care) (26).” For example, USPTO patent US20180275314A1 for “method and system for solar power forecasting” was linked to the micro-title of “solar thermal installer” and the task of “performing computer simulation of solar photovoltaic generation system performance or energy production to optimize efficiency”. Similarly, patent US2022083792A1 for a “method and device for providing data for creating a digital map” was linked to the micro-title “digital cartographer” and the task of “mapping forest tract data using digital mapping systems”. In addition to measuring automation (using our AII measure on occupation tasks) and augmentation (adapting our AII measure on micro-titles), we replicated the method proposed by Gmyrek et al. (36). This method uses the mean and standard deviation of task-level scores to distinguish between automation and augmentation (explained in “Beyond Automation: Measuring Augmentation” in Supplementary material).

## Supplementary Material

pgae320_Supplementary_Data

## Data Availability

The occupation data supporting this study’s findings are available from O*NET (47), at https://www.onetcenter.org/db_releases.html. The patent data are available in Google Patents Public Data (61) at https://console.cloud.google.com/marketplace/product/google_patents_public_datasets/google-patents-public-data, and can be retrieved using the query available in the project’s page. The Quarterly Census of Employment and Wages (QCEW) data are made available by the US Bureau of Labor Statistics at https://www.bls.gov/cew/downloadable-data-files.htm. All the datasets are compiled on the project’s page at https://social-dynamics.net/aii. Code necessary to reproduce the analyses in this study is available in the project’s page at https://social-dynamics.net/aii.

## Preprints

A preprint of this article is published at https://arxiv.org/pdf/2312.04714.
