# Supplementary material for: The potential impact of AI innovations on US occupations
Source: PNAS Nexus. 2024 Sep 24;3(9):pgae320. doi: 10.1093/pnasnexus/pgae320 (PMC11421150; doi:10.1093/pnasnexus/pgae320)
Supplement: pgae320_Supplementary_Data [file pgae320_supplementary_data.pdf]

Supplementary Material

Data

Data Collection

In a total of 24,758 AI patents granted between 2015 and 2022, the majority contained the keyword machine learning (46%), followed by the keyword neural network (32%), artificial intelligence (9%), and deep learning (6%) (Table S1).

Table S1. Number of patents based on keywords.

Keywords	Number of patents
machine learning	10904
neural network	9364
artificial intelligence	2674
deep learning	1848
planning	1050
natural language processing	917
reinforcement learning	506
computer vision	463
speech processing	126
predictive analytics	69
robotics	64
control methods	29
knowledge representation	24

From 759 occupations defined in O*NET, the number of tasks for each occupation ranges from 4 to 286, with a median of 20 tasks (Figure S1).

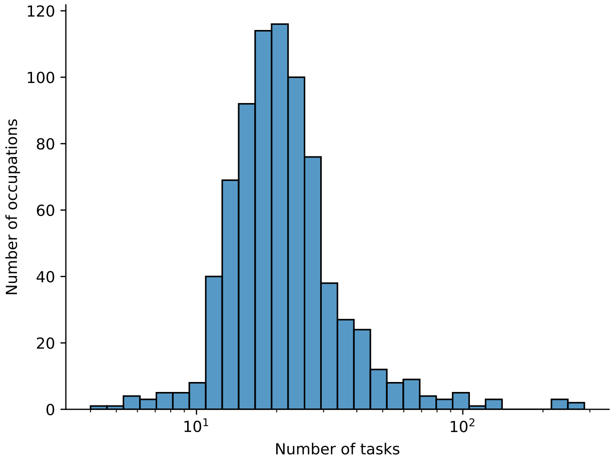

Fig. S1. The distribution of the number of tasks per occupation.

To compute SML scores, Brynjolfsson et al. [18] used a set of rubrics to guide crowdworkers to assess the suitability of tasks for machine learning (Table S2). Note that only one item in the rubrics is about interpersonal interactions.

Table S2. Rubrics to compute SML scores as developed by Brynjolfsson et al. [18].

No	Rubric
Q1	Information needed to complete the task (inputs) and outputs can be explicitly specified in machine-readable format
Q2	Task information is recorded or recordable by computer
Q3	Task does not require a wide range of complex outputs (mental and/or physical)
Q4	Task feedback (on the success of outputs) is immediate
Q5	The task output is error tolerant
Q6	It is not important that outputs are perceived to come from a human
Q7	Task does not require complex, abstract reasoning
Q8	Task is principally concerned with matching information to concepts, labels, predictions, or actions
Q9	Task does not require detailed, wide-ranging conversational interaction with a customer or other person
Q10	Task is highly routine and repeated frequently
Q11	Task is describable with rules
Q12	There is no need to explain decisions during task execution
Q13	Task can be converted to answering multiple choice questions, ranking alternative options, predicting a number, or grouping similar objects
Q14	Long term planning is not required to successfully complete the task
Q15	The task requires working with text data or might require working with text in the future
Q16	The task requires working with image/video data or might require working with image/video data in the future
Q17	The task requires working with speech data or might require working with speech data in the future
Q18	The task requires working with other types of data (other than text, image/video, and speech)
Q19	Many components of the task can be completed in a second or less
Q20	Each instance, completion, or execution of the task is similar to the other instances in how it is done and these actions can be measured
Q21	Actions in the task must be completed in a very specific order, and practicing the task to get better is easy

Table S3. Examples of occupational descriptions in O*NET.

Occupation Title	Sector	Description
Cardiovascular Technologists and Technicians	Healthcare	Conduct tests on pulmonary or cardiovascular systems of patients for diagnostic purposes. May conduct or assist in electrocardiograms, cardiac catheterizations, pulmonary functions, lung capacity, and similar tests. Includes vascular technologists.
Orthodontists	Healthcare	Examine, diagnose, and treat dental malocclusions and oral cavity anomalies. Design and fabricate appliances to realign teeth and jaws to produce and maintain normal function and to improve appearance.
Medical Records and Health Information Technicians	Healthcare	Compile, process, and maintain medical records of hospital and clinic patients in a manner consistent with medical, administrative, ethical, legal, and regulatory requirements of the health care system. Process, maintain, compile, and report patient information for health requirements and standards in a manner consistent with the healthcare industry's numerical coding system.
Numerical Tool and Process Control Programmers	Manufacturing	Develop programs to control machining or processing of metal or plastic parts by automatic machine tools, equipment, or systems.
Multi-Media Artists and Animators	Information	Create special effects, animation, or other visual images using film, video, computers, or other electronic tools and media for use in products or creations, such as computer games, movies, music videos, and commercials.
Magnetic Resonance Imaging Technologists	Healthcare	Operate Magnetic Resonance Imaging (MRI) scanners. Monitor patient safety and comfort, and view images of area being scanned to ensure quality of pictures. May administer gadolinium contrast dosage intravenously. May interview patient, explain MRI procedures, and position patient on examining table. May enter into the computer data such as patient history, anatomical area to be scanned, orientation specified, and position of entry.
Nuclear Medicine Technologists	Healthcare	Prepare, administer, and measure radioactive isotopes in therapeutic, diagnostic, and tracer studies using a variety of radioisotope equipment. Prepare stock solutions of radioactive materials and calculate doses to be administered by radiologists. Subject patients to radiation. Execute blood volume, red cell survival, and fat absorption studies following standard laboratory techniques.
Software Developers, Applications	Information	Develop, create, and modify general computer applications software or specialized utility programs. Analyze user needs and develop software solutions. Design software or customize software for client use with the aim of optimizing operational efficiency. May analyze and design databases within an application area, working individually or coordinating database development as part of a team. May supervise computer programmers.
Electro-Mechanical Technicians	Manufacturing	Operate, test, maintain, or calibrate unmanned, automated, servo-mechanical, or electromechanical equipment. May operate unmanned submarines, aircraft, or other equipment at worksites, such as oil rigs, deep ocean exploration, or hazardous waste removal. May assist engineers in testing and designing robotics equipment.
Industrial Truck and Tractor Operators	Manufacturing	Operate industrial trucks or tractors equipped to move materials around a warehouse, storage yard, factory, construction site, or similar location.

To conduct the thematic analysis on industry sectors, we used the occupation titles and descriptions in O*NET. For brevity, we provide 10 examples in Table S3 and we refer the reader to the full list in O*NET [54].

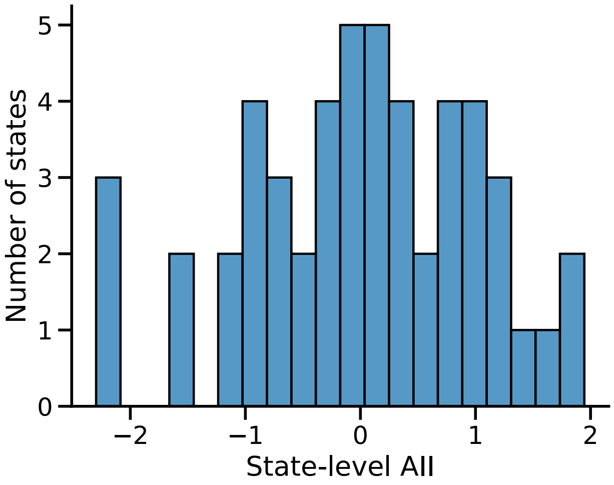

Fig. S2. Frequency distribution plots of the AII measure at the regional level.

Summary Statistics

We checked our AII measure at state-level distribution to ensure that it was normally distributed before using it in our regional-level analysis.

Task-Patent Matching

Validating the similarity metric used for matching

To ensure the specificity of our task-patent matching method, we plotted the mean and max of the AII measure. We empirically found that by using the max, we retained the most relevant patent (Figure S3). To further validate our task-patent matching method, we manually annotated a random sample of 100 task-patent pairs in terms of task-patent relevance; two of the authors who did the annotations achieved a Cohen's kappa of 0.84 (Kappa values in the [0.81, 1] range suggest almost perfect agreement).

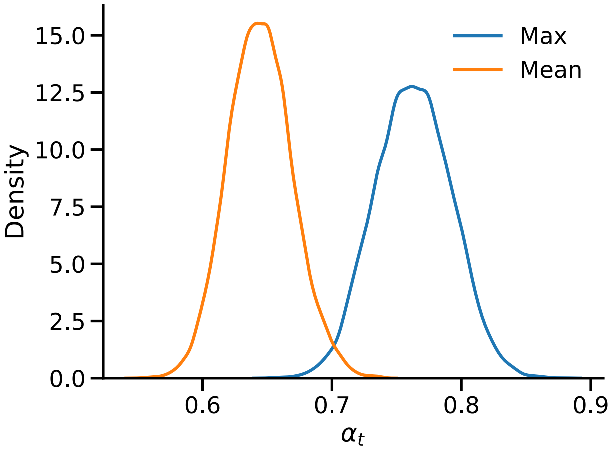

Fig. S3. Distributions of the task-patent similarity based on different aggregations. By focusing only on the most relevant patents, we captured the specificity of the AI impact measurement for each task, reducing noise, and incorporating a wider range of AI-related advancements.

Task-patent matching using multi-instance learning

As an alternative to our AII measure for task-patent matching, we implemented a multi-instance learning approach. To do so, we followed a seven-step procedure:

Step 1. Selection of Data: We selected 100 patent abstracts, each related to just one specific task, and ensured that every abstract was closely linked to the task it described. **Step 2.**

Sentence Segmentation: We chunked these abstracts into individual sentences, resulting in a total of 356 sentences.

Step 3. Embedding Generation for Sentences: We then generated embeddings for each of the individual sentences.

Step 4. Embedding Generation for Tasks: Simultaneously, we generated embeddings for the tasks associated with patent abstracts selected in Step 1. This parallel embedding process ensured that both the tasks and the patent sentences were represented in a comparable semantic space.

Step 5. Pairwise Similarity Calculation: With these embeddings at hand, we then computed the pairwise similarity between each task and all sentences from the abstracts related to it. This step involved calculating the cosine similarity between embeddings, which quantifies how semantically close two pieces of text are.

Step 6. Maximum of Similarity Values: For each task, we selected the maximum similarity value from the set of pairwise similarities. This value represents the closest semantic match between the task and sentences in the patent abstracts.

Step 7. Comparison of Similarities: Finally, we compared these maximum sentence-level similarity values with the similarity values obtained from whole abstract embeddings. This comparison allowed us to determine whether analyzing sentences individually provides a better match to the tasks than analyzing whole abstracts.

Following this seven-step procedure, we found a high correlation between similarity scores computed using embeddings of the entire abstract and those using embeddings of individual abstract sentences ($\rho = 0.85$). Moreover, 99 of the 100 selected tasks showed a higher similarity score with whole-abstract embeddings compared to the best-matching sentence. This suggests that using the entire abstract provides more context and leads to better matches than using the multi-instance learning approach.

Table S4. Example task-patent pairs matched with AII. The dash (-) sign means that the patent is the same as the cell above it.

Occupation	Sector	Task	Matching Patent Title	Matching Patent Abstract	Similarity
Substance Abuse and Behavioral Disorder Counselors	Healthcare	Develop client treatment plans based on research, clinical experience, and client histories.	Methods and systems for radiotherapy treatment planning	Example methods for radiotherapy treatment planning are provided. One example method may include obtaining training data that includes multiple treatment plans associated with respective multiple past patients; [...]	0.8018
Mental Health Counselors	Healthcare	Develop and implement treatment plans based on clinical experience and knowledge.	-	-	0.8082
Radiation Therapists	Healthcare	Administer prescribed doses of radiation to specific body parts, using radiation therapy equipment according to established practices and standards.	-	-	0.8330
Architectural and Civil Drafters	Manufacturing	Reproduce drawings on copy machines or trace copies of plans and drawings, using transparent paper or cloth, ink, pencil, and standard drafting instruments.	Line drawing generation	Computing systems and computer-implemented methods can be used for automatically generating a digital line drawing of the contents of a photograph. [...] The training data set teaches the neural network to trace the edges and features of objects in the photographs, as well as which edges or features can be ignored. [...]	0.8038
Multi-Media Artists and Animators	Information	Create pen-and-paper images to be scanned, edited, colored, textured, or animated by computer.	-	-	0.8396
Detectives and Criminal Investigators	Public administration	Create sketches and diagrams by hand or with computer software to depict crime scenes.	-	-	0.8195
Photographic Process Workers and Processing Machine Operators	Other services	Operate scanners or related computer equipment to digitize negatives, photographic prints, or other images.	-	-	0.8167
Software Developers, Applications	Information	Consult with customers about software system design and maintenance.	System and method for custom-fitting services to consumer requirements	Systems and methods for custom-fitting a service solution to consumer requirements are provided. [...]	0.8020
Child, Family, and School Social Workers	Education	Interview clients individually, in families, or in groups, assessing their situations, capabilities, and problems to determine what services are required to meet their needs.	-	-	0.8010
Solar Photovoltaic Installers	Construction	Determine photovoltaic (PV) system designs or configurations based on factors such as customer needs, expectations, and site conditions.	-	-	0.8031

Matching abstracts (not only titles)

Patents tend to be general, and specific tasks may not be mentioned simply in the patent title. To address this, our method uses the patent abstract that is richer in text and more likely to reference a specific task or even entire sector(s) (Table S4). As a result, we were able to match the same patent with occupations in multiple sectors. For example, a patent that provides methods for radiotherapy treatment planning not only matches the task of radiation therapists, but also counselors (abuse and behavioral disorder) who need treatment plans. Another example is a patent for custom-fitting a service solution to consumer requirements that is applicable to occupations from different sectors (e.g., software developers, social workers, and solar photovoltaic installers).

Table S5. List of the the tasks for the “elevator installers and repairers” occupation, and whether each task is impacted or not based on AII. A task is impacted when there is a patent with a similarity exceeding the 90th percentile threshold.

Task	Is impacted?
Locate malfunctions in brakes, motors, switches, and signal and control systems, using test equipment.	Yes
Assemble, install, repair, and maintain elevators, escalators, moving sidewalks, and dumbwaiters, using hand and power tools, and testing devices such as test lamps, ammeters, and voltmeters.	No
Test newly installed equipment to ensure that it meets specifications, such as stopping at floors for set amounts of time.	No
Check that safety regulations and building codes are met, and complete service reports verifying conformance to standards.	No
Connect electrical wiring to control panels and electric motors.	No
Adjust safety controls, counterweights, door mechanisms, and components such as valves, ratchets, seals, and brake linings.	No
Read and interpret blueprints to determine the layout of system components, frameworks, and foundations, and to select installation equipment.	No
Inspect wiring connections, control panel hookups, door installations, and alignments and clearances of cars and hoistways to ensure that equipment will operate properly.	No
Disassemble defective units, and repair or replace parts such as locks, gears, cables, and electric wiring.	No
Maintain log books that detail all repairs and checks performed.	No
Participate in additional training to keep skills up to date.	No
Attach guide shoes and rollers to minimize the lateral motion of cars as they travel through shafts.	No
Connect car frames to counterweights, using steel cables.	No
Bolt or weld steel rails to the walls of shafts to guide elevators, working from scaffolding or platforms.	No
Assemble elevator cars, installing each car’s platform, walls, and doors.	No
Install outer doors and door frames at elevator entrances on each floor of a structure.	No
Install electrical wires and controls by attaching conduit along shaft walls from floor to floor and pulling plastic-covered wires through the conduit.	No
Cut prefabricated sections of framework, rails, and other components to specified dimensions.	No
Operate elevators to determine power demands, and test power consumption to detect overload factors.	No
Assemble electrically powered stairs, steel frameworks, and tracks, and install associated motors and electrical wiring.	No

Previous Attempts to Link Tasks to Patents

Word-matching methods linking tasks to patents. A word-matching method employs a dictionary approach, parsing task descriptions to identify verb-noun pairs associated with each task [62]. This approach captures the task essence concisely and specifically, such as the pair “install, sensor”, offering a clear representation of the task. Using the same method, AI patent titles are also processed to extract verb-noun pairs describing the tasks targeted by each patent. The relative frequency of similar pairs in tasks and patent titles determines the AI exposure score, indicating the level of task exposure to AI.

Word-matching methods: Potential false positives. Webb method [62] identified “elevator installers” as one of the most impacted occupations by AI. However, he also acknowledged that this classification is an example of false positives caused by word matching methods being coarse-grained. By applying our deep-learning method instead, we learn that nearly all the tasks of the occupation “elevator installers” are indeed not impacted (Table S5): only 1 out of 20 tasks was impacted. This speaks to the robustness of a deep-learning approaches compared to a word-matching approach.

Table S6. List of tasks with zero AI Exposure Score, indicating no associated patents [62]. In contrast, AII identifies relevant patents for those tasks.

Task	Extracted pairs	AI exposure score	Most Similar Patent Abstract	Similarity
Document and maintain records of precision agriculture information.	(maintain, record)	0.000	A method and system for predicting soil and/or plant condition in precision agriculture with a classification of measurement data for providing an assignment of a measurement parcel to classes of interest. The assignment is used for providing action recommendations, particularly in real time or close to real time, to a farmer and/or to an agricultural device based on acquired measurement data, particularly remote sensing data, and wherein a classification model is trained by a machine learning algorithm, e.g. relying on deep learning for supervised and/or unsupervised learning, and is potentially continuously refined and adapted thanks to a feedback procedure.	0.790
Apply precision agriculture information to specifically reduce the negative environmental impacts of farming practices.	(apply, information)	0.000	A method and system for predicting soil and/or plant condition in precision agriculture with a classification of measurement data for providing an assignment of a measurement parcel to classes of interest. The assignment is used for providing action recommendations, particularly in real time or close to real time, to a farmer and/or to an agricultural device based on acquired measurement data, particularly remote sensing data, and wherein a classification model is trained by a machine learning algorithm, e.g. relying on deep learning for supervised and/or unsupervised learning, and is potentially continuously refined and adapted thanks to a feedback procedure.	0.813
Install, calibrate, or maintain sensors, mechanical controls, GPS-based vehicle guidance systems, or computer settings.	(maintain, sensor)	0.000	A vehicle computing system validates location data received from a Global Navigation Satellite System receiver with other sensor data. In one embodiment, the system calculates velocities with the location data and the other sensor data. The system generates a probabilistic model for velocity with a velocity calculated with location data and variance associated with the location data. The system determines a confidence score by applying the probabilistic model to one or more of the velocities calculated with other sensor data. In another embodiment, the system implements a machine learning model that considers features extracted from the sensor data. The system generates a feature vector for the location data and determines a confidence score for the location data by applying the machine learning model to the feature vector. Based on the confidence score, the system can validate the location data. The validated location data is useful for navigation and map updates.	0.817

Word-matching methods: Potential false negatives. In addition to, at times, matching incorrectly tasks and patents (false positives), word matching methods may also fail to return genuine matches (false negatives). For example, Webb's paper reported a variety of tasks for which no patent was found (Table S6). However, by applying AII to those tasks, we instead found that there are indeed matching patents. This discrepancy likely arises from word-matching methods discarding multiple verb-noun pairs, and overlooking details in patent abstracts.

Most- and Least-Impacted Occupations and Industry Sections

Between 2010 and 2020

We computed the AII measure on occupations and industry sectors in a larger time window between 2010 and 2020. Similarly, we observed that the most impacted occupations come primarily from healthcare, information technology, and manufacturing, whereas the least impacted ones come from finance and insurance, education, and construction.

Table S7. 20 most- and least-impacted occupations ranked by the AII (Artificial Intelligence Impact) measure using AI patents from 2010 to 2020. For the 20 most- and least-impacted occupations, only 1 entry differs for each. This is because patents in 2015-2020 account for 96% of the total patents in 2010-2020.

Rank	Most-impacted	Least-impacted
1	Orthodontists	Pile-Driver Operators
2	Cardiovascular Technologists and Technicians	Graders and Sorters, Agricultural Products
3	Medical Records and Health Information Technicians	Floor Sanders and Finishers
4	Multi-Media Artists and Animators	Aircraft Cargo Handling Supervisors
5	Electro-Mechanical Technicians	Insurance Appraisers, Auto Damage
6	Magnetic Resonance Imaging Technologists	Insurance Underwriters
7	Nuclear Medicine Technologists	Reinforcing Iron and Rebar Workers
8	Software Developers, Applications	Farm Labor Contractors
9	Industrial Truck and Tractor Operators	Water and Liquid Waste Treatment Plant and System Operators
10	Numerical Tool and Process Control Programmers	Animal Scientists
11	Sound Engineering Technicians	Brokerage Clerks
12	Computer Programmers	Insulation Workers, Floor, Ceiling, and Wall
13	Life, Physical, and Social Science Technicians, All Other	Locomotive Firers
14	Earth Drillers, Except Oil and Gas	Management Analysts
15	Medical Transcriptionists	Podiatrists
16	Airline Pilots, Copilots, and Flight Engineers	Cooks, Short Order
17	Commercial Pilots	Shipping, Receiving, and Traffic Clerks
18	Physical Scientists, All Other	Helpers–Painters, Paperhangers, Plasterers, and Stucco Masons
19	Computer-Controlled Machine Tool Operators, Metal and Plastic	Team Assemblers
20	Biomedical Engineers	Ambulance Drivers and Attendants, Except Emergency Medical Technicians

Between 2010 and 2022

We computed the AII measure on occupations and industry sectors in a larger time window between 2010 and 2022. In this extended timeframe, we again observed that the most impacted occupations primarily come from healthcare, information technology, and manufacturing. Additionally, we noted an increasing impact of AI in scientific occupations.

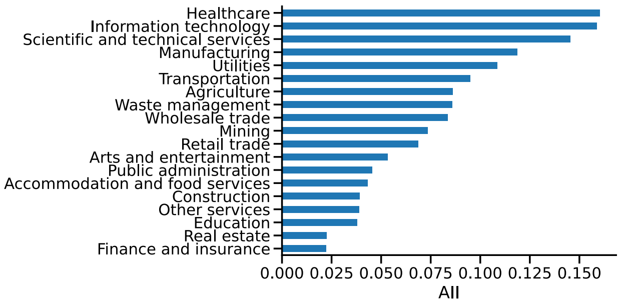

Fig. S4. Overall AI impact on industry sectors from 2010-2020, ranked from highest to lowest sector-level AII scores. A sector-level AII score represents the mean AII across all occupations within that sector. The only difference from the 2015-2020 version is the ordering of other services and construction sectors.

Table S8. 20 most- and least-impacted occupations ranked by the AII (Artificial Intelligence Impact) measure using AI patents from 2010 to 2022.

Rank	Most-impacted	Least-impacted
1	Cardiovascular Technologists and Technicians	Pile-Driver Operators
2	Sound Engineering Technicians	Graders and Sorters, Agricultural Products
3	Nuclear Medicine Technologists	Floor Sanders and Finishers
4	Magnetic Resonance Imaging Technologists	Aircraft Cargo Handling Supervisors
5	Air Traffic Controllers	Insurance Underwriters
6	Orthodontists	Reinforcing Iron and Rebar Workers
7	Electro-Mechanical Technicians	Farm Labor Contractors
8	Power Distributors and Dispatchers	Rock Splitters, Quarry
9	Industrial Truck and Tractor Operators	Brokerage Clerks
10	Police, Fire, and Ambulance Dispatchers	Locomotive Firers
11	Security Guards	Management Analysts
12	Physical Scientists, All Other	Podiatrists
13	Machinists	Cooks, Short Order
14	Atmospheric and Space Scientists	Shipping, Receiving, and Traffic Clerks
15	Computer-Controlled Machine Tool Operators, Metal and Plastic	Proofreaders and Copy Markers
16	Medical Records and Health Information Technicians	Helpers–Painters, Paperhangers, Plasterers, and Stucco Masons
17	Textile Knitting and Weaving Machine Setters, Operators, and Tenders	Team Assemblers
18	Radio Operators	Political Scientists
19	Medical Transcriptionists	Paralegals and Legal Assistants
20	Physician Assistants	Telemarketers

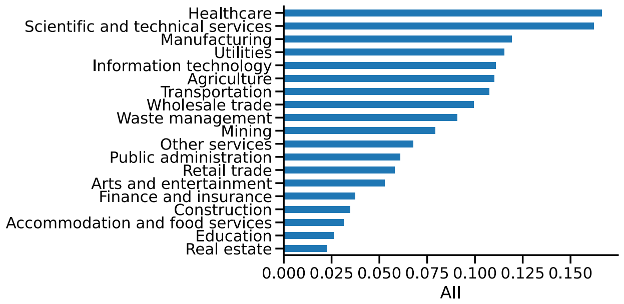

Fig. S5. Overall AI impact on industry sectors from 2010-2022, ranked from highest to lowest sector-level AII scores. A sector-level AII score represents the mean AII across all occupations within that sector.

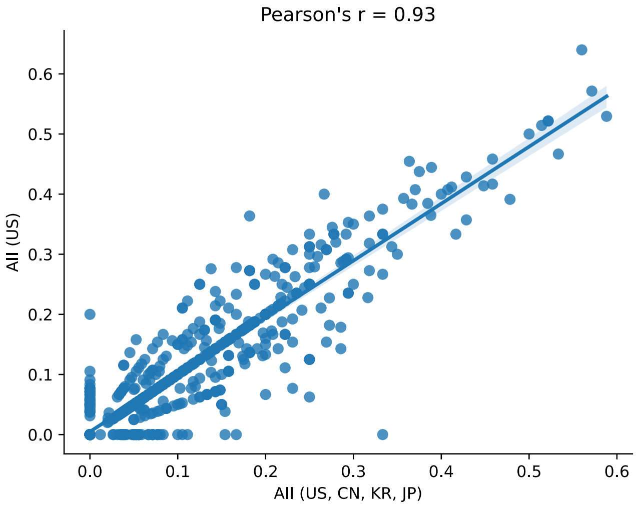

Fig. S6. Correlation between AII calculated with only US patents and with patents from US, China, and Japan.

Using patents from the U.S., China, Japan, and Korea

We collected all 153,854 patents from China, Japan, and South Korea written in English from Google Patents Public Data during the period of study. The AI patents from these countries and the U.S. make up 81% of the total published AI patents.

We created two sets that contain patents from: *(1)* U.S. only; and *(2)* U.S., China, Japan, and Korea. As seen in Figure S6, the AII scores computed on the two sets of patents have a correlation of $r = 0.93$. With the newly added patents from China, Japan, and Korea, additional occupations potentially impacted include "bakers", "solar photovoltaic installers", and "dredge operators", predominantly affected by patents originating from China.

Job Vacancy Rates by Sector *vs.* sector-level AII

AII at sector level and vacancy rates are positively correlated, with a Pearson's correlation coefficient of $r = 0.28$ ($p = 0.29$) but not statistically significant (Figure S7).

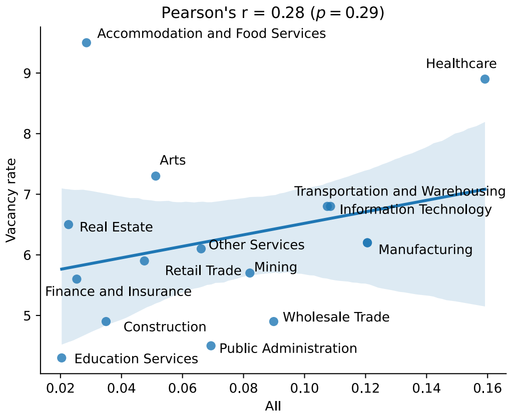

Fig. S7. Job vacancy rates by sector vs. sector-level AII with all industry sectors.

Impact of Robots and Software

We investigated the relationship between the adapted AII score for robots and software and changes in wages and employment between 1980 and 2010. We did so by following Webb's methodology [62], and by using census data and patents related to robots or software.

Data

For robot-related patents, we filtered patents based on whether the keywords "robot" or "manipulat" appeared in the patents' titles and abstracts. Additionally, we ensured the resulting set of patents did not have Cooperative Patent Classification (CPC) codes A61 (medical or veterinary science; hygiene) or B01 (physical or chemical processes or apparatus in general), following Webb's methodology [62]. For software-related patents, our filter included any patent with the keywords "software", "computer", or "program", while excluding any that mentioned "chip", "semiconductor", "bus", "circuity", or "circuitry".

To compute the adapted AII score for either robots or software, we applied the same formula (Equation 1) used to compute the impact of AI at the task level but replaced the set of patents with those associated either with robots or software. To compute the impact of robots or software at the occupation level, we used the same formula (Equation 2) because it depends on the number of tasks that are impacted rather than the patents themselves.

For changes in wages and employment, we used individual-level microdata from the US Census between 1960 and 2000 and from the ACS between 2000 and 2018, both of which were provided in the Integrated Public Use Microdata Series (IPUMS) [56]. We restricted the analysis to individuals aged between 18 and 65 who were employed and engaged in some form of work.[1] We calculated the average wages and the proportion of hours worked within each industry-occupation pair (referred to as an industry-occupation cell). We used a number of additional census control variables including age, gender, level of education, and offshorability (i.e., the degree to which an occupation requires either direct interpersonal interaction or proximity to a specific work location). The measure of offshorability was developed by [32] and standardized by [12]. For all the subsequent analyses, we used the IPUMS "occ1990" occupational classification and "ind1990" industry classification.

To ensure that the relationship between the AII scores for robots and software and changes in employment and wages is measured proportionately to the workforce size, we applied a number of controls following Webb's methodology [62]. First, we applied the IPUMS survey's individual weight (PERWT) adjusted by the proportion of full-time work in each industry and occupation combination, resulting into a "labor-supply weight". This adjustment yields the total number of full-time-equivalent (FTE) employees in each industry-occupation pair. Additionally, we introduced a "demographic-adjusted" labor-supply weight to reflect demographic shifts since 1980. This involved categorizing demographic groups by gender, race (black, white, other), level of education (less than high school, high school graduate, some college, bachelor's degree), and age group (in five-year intervals). The adjusted weight for 2010 was then computed by maintaining each group's proportionate weight from 1980 (that is, adjusting each data point by the 1980 to 2010 weight ratio for its demographic group). For wage calculations, we determined the real weekly wages (in 2016 dollars) for 1980 and 2010, focusing on full-time-full-year (FTFY, over 35 hours/week and 40 weeks/year) employees, applying a 98% winsorization to control for extreme values in the earnings data annually. We then aggregated the census data into industry-occupation-year groups using the "ind1990" and "occ1990" classifications from IPUMS. In our subsequent regression analyses, the education variable was adjusted according to the labor-supply-weighted average years of education for each industry-occupation cell in 1980, categorized into terciles.

Regression Model

To measure the relationship between the adapted AII score for robots and software and changes in employment and wages, we estimated variations of the following regression:

$$\Delta y_{o,i,t} = \alpha_i + \beta x_o + \gamma Z_o + \varepsilon_{o,i,t} \quad (5)$$

The unit of observation is an occupation-industry-year cell, such as welders in auto manufacturing in 1980, with o denoting occupation, i industry, and t year. The dependent variable is the difference between 1980 and 2010 of an outcome variable of interest (i.e., employment and wages). On the right-hand side (Equation 5), we included industry fixed effects α_i; the exposure of the occupation to robots or software x_o; and the vector of controls Z_o contains occupation-level variables such as terciles of average years of education and offshorability.

To measure the change in employment, we used the DHS changes, also known as arc percentage change or percent change relative to the midpoint. DHS is a symmetric measure of the growth rate defined as the difference between two values divided by their average [27]. This results in a second-order approximation of the log change for growth rates near zero; values are restricted to being between -2 and 2, with -2 and 2 representing exit and entry respectively.

To measure the change in wages, we used the log change in real weekly wages for full-time and full-year workers in each industry-occupation cell. For the adapted AII scores, we transformed the raw scores to be in employment-weighted percentiles. Thus, a score of 90 means that 10% of workers work in occupations with a higher exposure. To construct the industry-occupation cell and account for industry fixed effects, we used the IPUMS "ind1990" industry classification code. Finally, the sample used to measure the relationship between exposure to robots and changes in employment and wages is restricted to industries within the manufacturing sector.

Results

The models show that moving from the 25th to the 75th percentile of exposure to robots is associated with a decline in wages between 2 and 4% (Table S9), depending on the specification, and a varying effect in industry employment share of between -9 and 18% (Table S10). Similarly, moving from the 25th to the 75th percentile of exposure to software is associated with a decline in wages of between 4 and 7% (Table S11), and a decline in within-industry employment

[1] We used the field WORKEDYR from the IPUMS data, which indicates whether the person had worked at all for profit, pay, or as an unpaid family worker during the previous year. For the census samples, the reference period is the previous calendar year; for the ACS and the PRCS, the reference period is the preceding 12 months.

Table S9. Change in wages vs. exposure to robots, 1980-2010. Each observation is an occupation-industry cell. Dependent variable is 100x change in log wage between 1980 and 2010, winsorized at the top and bottom 1%. Education variables are terciles of average years of education for occupation-industry cells in 1980. Wages are cells' mean weekly wage for full-time, full-year workers in 1980. Offshorability is an occupation-level measure from Autor and Dorn (2013). Observations are weighted by cell's labor supply, averaged between 1980 and 2010.

	(1)	(2)	(3)	(4)	(5)	(6)
AII	-0.076***	-0.076***	-0.076***	-0.035*	-0.042**	
	(0.018)	(0.018)	(0.018)	(0.018)	(0.018)	
Offshorability			-2.029***	-0.693	5.077***	5.162***
			(0.701)	(0.713)	(0.730)	(0.729)
Medium education				5.271***	9.529***	9.756***
				(0.962)	(0.941)	(0.937)
High education				8.732***	27.022***	27.503***
				(0.984)	(1.276)	(1.261)
Wage					-0.056***	-0.056***
					(0.003)	(0.003)
Wage squared					0.000***	0.000***
					(0.000)	(0.000)
Adjusted R^2	0.003	0.036	0.037	0.049	0.126	0.125
Industry FEs		✓	✓	✓	✓	✓
Observations	5957	5957	5957	5957	5957	5957

Notes: *p<0.1; **p<0.05; ***p<0.01

shares of between 3 and 10% (Table S12). Recall that these are within-industry effects. Therefore, these results do not simply say that manufacturing jobs are exposed to robots, and manufacturing has (for other reasons) declined. Rather, they show that within each manufacturing industry, the particular occupations exposed to robots have declined much more than those that are not exposed.

However, these relationships might be influenced by external factors such as offshorability, industry effects, educational levels, and wage polarization. Given the emergence of offshoring as a significant trend between 1980 and 2010, we incorporated it as a control variable in our analysis. Additionally, we anticipated that industry-specific effects would capture variations stemming from trade dynamics and evolving consumer preferences impacting product demand. Another consideration was the significant demographic and skill shifts observed between 1980 and 2010. It is plausible therefore that the workforce's educational enhancement, particularly the increase in highly skilled individuals, altered the labor supply dynamics, diminishing the availability of low-skilled workers who are most vulnerable to automation. This shift could mislead us into attributing the decrease in low-skilled labor demand to automation, overlooking the actual supply reduction. Finally, the potential of wage polarization, not directly linked to automation, is another factor to control for because previous economic studies have shown a decline in middle-skill workers' wages within the timeframe of our study, contrasting with a rise in top-tier salaries [49]. Despite accounting for these factors, the relationship between exposure to robots or software and changes in employment or wages remained statistically significant.

Table S10. Change in employment vs. exposure to robots, 1980-2010. Each observation is an occupation-industry cell. Dependent variable is 100x DHS change of a cell's share of overall employment between 1980 and 2010, winsorized at the top and bottom 1%. Education variables are terciles of average years of education for occupation-industry cells in 1980. Wages are cells' mean weekly wage for full-time, full-year workers in 1980. Offshorability is an occupation-level measure from Autor and Dorn (2013). Observations are weighted by cell's labor supply, averaged between 1980 and 2010.

	(1)	(2)	(3)	(4)	(5)	(6)
AII	0.360***	0.373***	0.360***	-0.124	-0.188**	
	(0.064)	(0.063)	(0.063)	(0.080)	(0.079)	
Offshorability			4.639*	0.571	15.211***	15.233***
			(2.514)	(2.535)	(2.651)	(2.652)
Medium Education				-29.584***	-20.301***	-15.843***
				(3.916)	(3.885)	(3.408)
High Education				-40.941***	6.256	12.004***
				(4.296)	(5.180)	(4.588)
Wage					-0.113***	-0.113***
					(0.012)	(0.012)
Wage squared					0.000***	0.000***
					(0.000)	(0.000)
Adjusted R^2	0.005	0.067	0.067	0.082	0.119	0.118
Industry FEs		✓	✓	✓	✓	✓
Observations	5957	5957	5957	5957	5957	5957

Note: *p<0.1; **p<0.05; ***p<0.01

Table S11. Change in wages vs. exposure to software, 1980-2010. Each observation is an occupation-industry cell. Dependent variable is 100x change in log wage between 1980 and 2010, winsorized at the top and bottom 1%. Education variables are terciles of average years of education for occupation-industry cells in 1980. Wages are cells' mean weekly wage for full-time, full-year workers in 1980. Offshorability is an occupation-level measure from Autor and Dorn (2013). Observations are weighted by cell's labor supply, averaged between 1980 and 2010.

	(1)	(2)	(3)	(4)	(5)	(6)
AII	-0.141***	-0.131***	-0.135***	-0.068**	-0.077***	
	(0.030)	(0.030)	(0.030)	(0.031)	(0.029)	
Offshorability			-3.498***	0.105	14.785***	15.036***
			(1.106)	(1.150)	(1.133)	(1.129)
Medium education				9.695***	24.125***	24.380***
				(1.678)	(1.617)	(1.614)
High education				19.347***	73.521***	74.252***
				(1.743)	(2.051)	(2.033)
Wage					-0.170***	-0.171***
					(0.005)	(0.005)
Wage squared					0.000***	0.000***
					(0.000)	(0.000)
Adjusted R^2	0.001	0.028	0.028	0.035	0.137	0.136
Industry FEs		✓	✓	✓	✓	✓
Observations	18724	18724	18724	18724	18724	18724

Note: *p<0.1; **p<0.05; ***p<0.01

Table S12. Change in employment vs. exposure to software, 1980-2010. Each observation is an occupation-industry cell. Dependent variable is 100x DHS change of a cell's share of overall employment between 1980 and 2010, winsorized at the top and bottom 1%. Education variables are terciles of average years of education for occupation-industry cells in 1980. Wages are cells' mean weekly wage for full-time, full-year workers in 1980. Offshorability is an occupation-level measure from Autor and Dorn (2013). Observations are weighted by cell's labor supply, averaged between 1980 and 2010.

	(1)	(2)	(3)	(4)	(5)	(6)
AII	-0.126***	-0.063	-0.057	-0.196***	-0.195***	
	(0.042)	(0.040)	(0.040)	(0.040)	(0.040)	
Offshorability			5.616***	-2.606*	4.628***	5.263***
			(1.452)	(1.500)	(1.550)	(1.545)
Medium education				-29.554***	-22.455***	-21.809***
				(2.188)	(2.212)	(2.209)
High education				-43.686***	-17.769***	-15.918***
				(2.273)	(2.807)	(2.783)
Wage					-0.093***	-0.094***
					(0.007)	(0.007)
Wage squared					0.000***	0.000***
					(0.000)	(0.000)
Adjusted R^2	0.000	0.114	0.115	0.132	0.146	0.145
Industry FEs		✓	✓	✓	✓	✓
Observations	18724	18724	18724	18724	18724	18724

Note: *p<0.1; **p<0.05; ***p<0.01

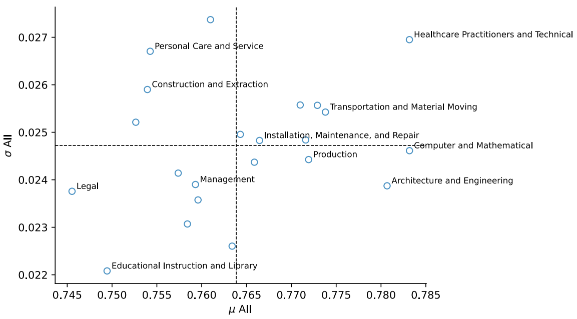

Fig. S8. Automation *vs.* augmentation potential computed using the mean and standard deviation of similarity scores as per [36] (bottom-left: not impacted; top-left: augmented; top-right: the big unknown; bottom-right: automated).

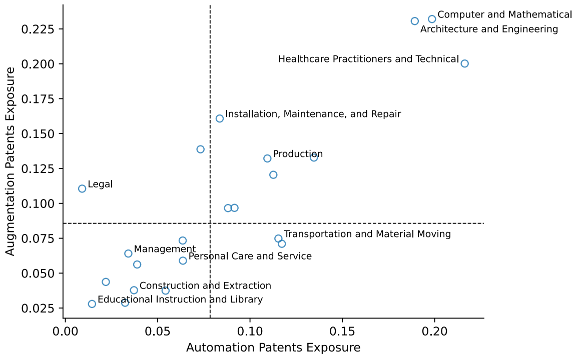

Fig. S9. Automation *vs.* augmentation using patent similarity to tasks and micro-titles defined in the Census Alphabetical Index of Occupations and Industries (CAI) [11].

Beyond Automation: Measuring Augmentation

Figure S8 shows the results as per Gmyrek *et al.* [36]'s method. To compare Autor *et al.*'s [11] and Gmyrek *et al.*'s [36] methods, for each occupation group defined by the first two digits—coarser-grained classification compared to the six digits classification—of the Standard Occupational Classification (SOC) code (`https://www.bls.gov/soc/socguide.htm`), we computed the average similarity value for automation and augmentation. We then took the median of the two average values and created a quadrant (automation vs. augmentation). The top left quadrant then indicates occupations that are likely to be exposed to augmentation, while the bottom right quadrant shows the exposure to automation. Figure S9 shows the results as per Autor et al [11]'s method, adapted to be visually similar with that of Figure S8. From these two figures, we observed that, in line with our results, Gmyrek *et al.* [36]'s method identified personal care (e.g., hearing aid specialist), construction (e.g., electrical and electronics repairers), and food preparation (e.g., food science technician) occupations as more exposed to augmentation than automation.
